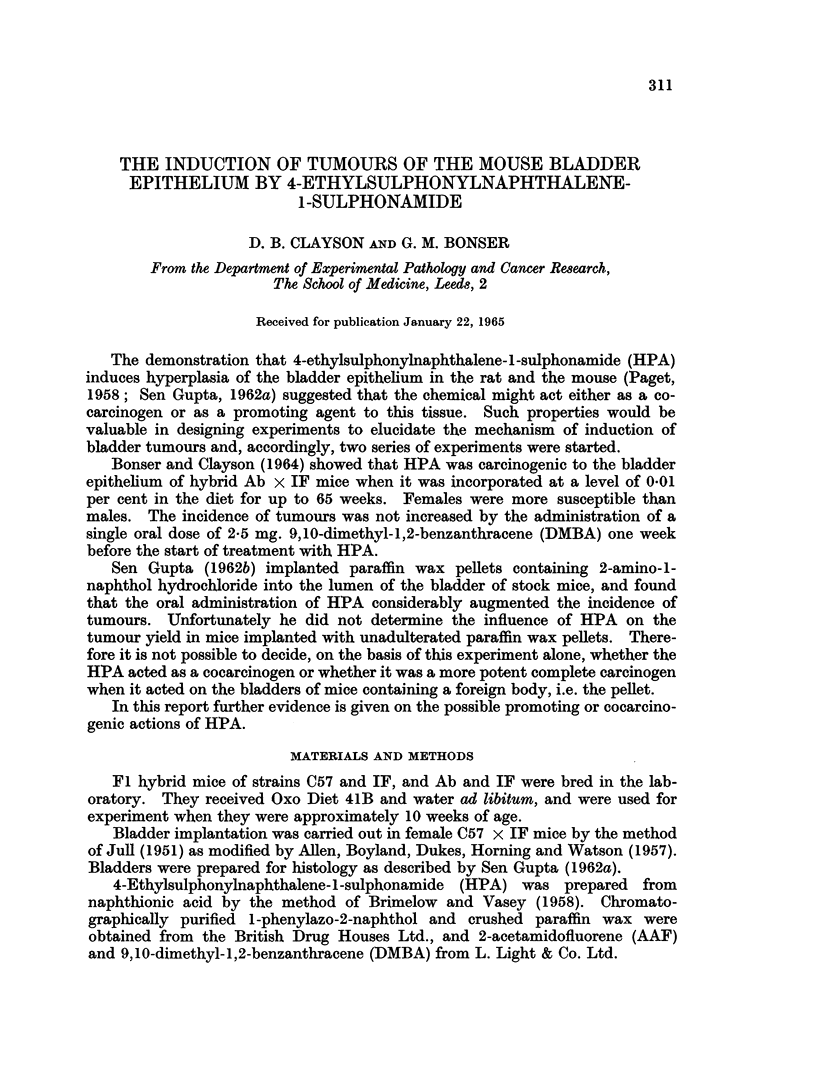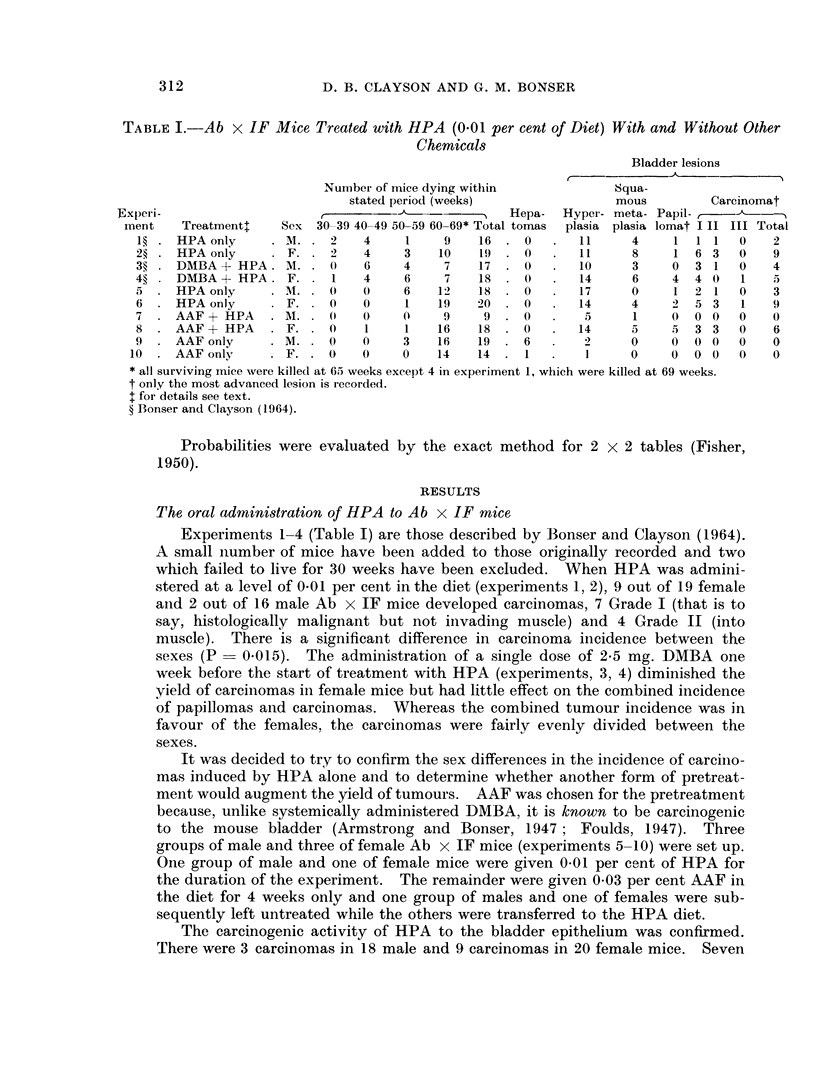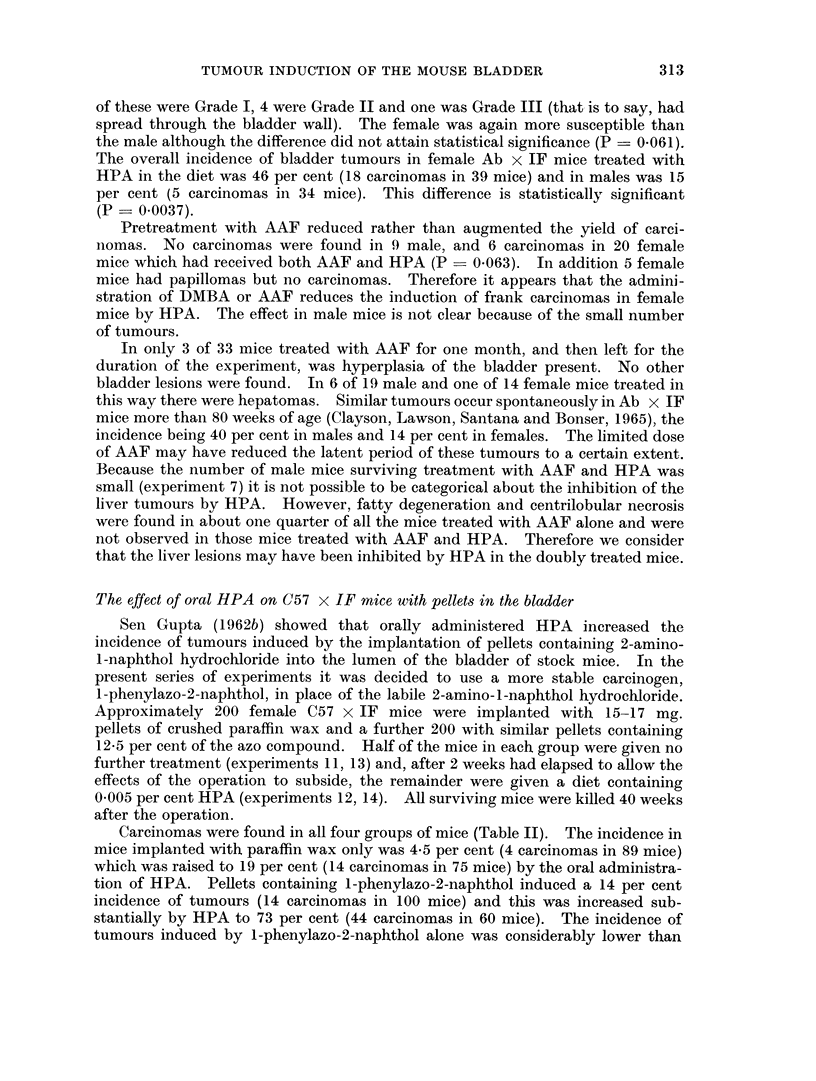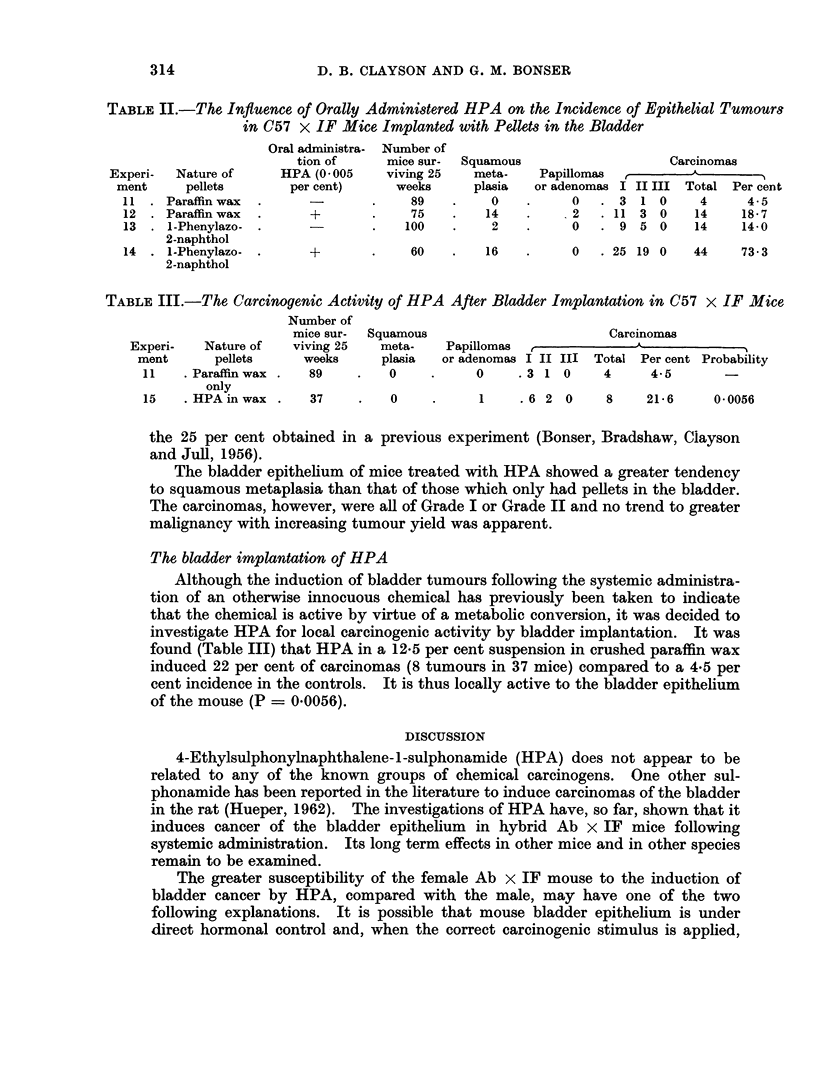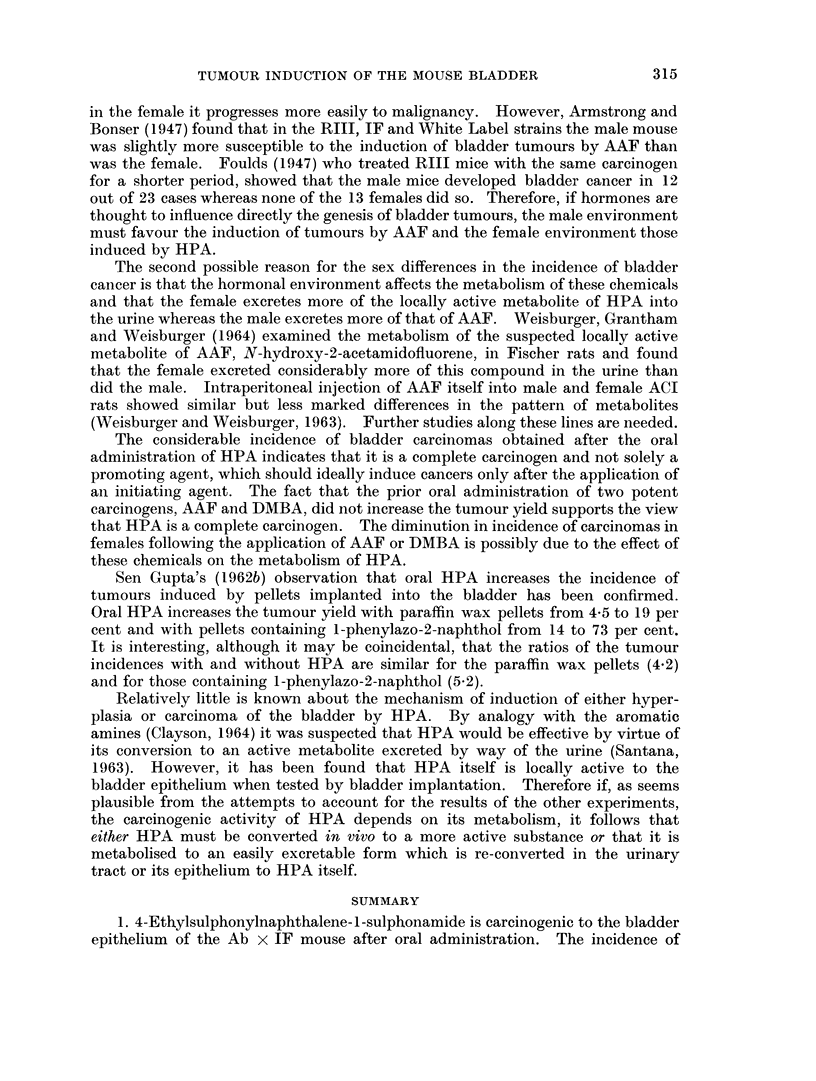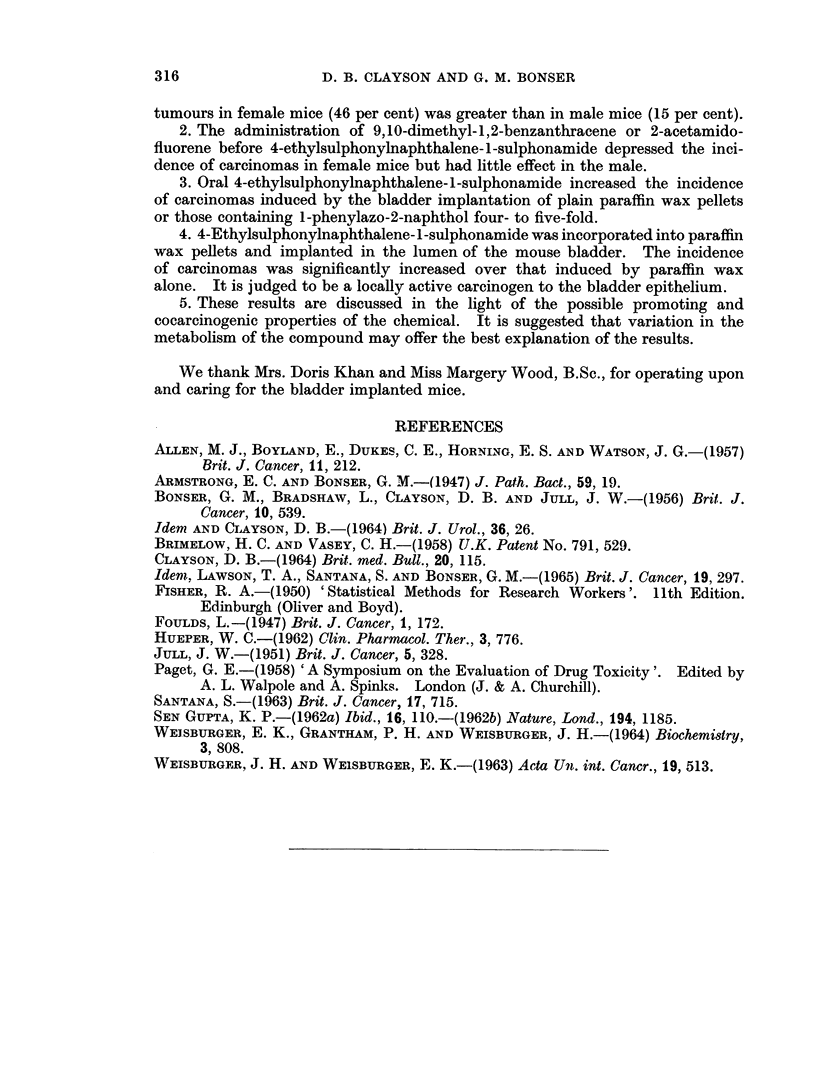# The Induction of Tumours of the Mouse Bladder Epithelium by 4-Ethylsulphonylnaphthalene-1-Sulphonamide

**DOI:** 10.1038/bjc.1965.35

**Published:** 1965-06

**Authors:** D. B. Clayson, G. M. Bonser


### THE INDUCTION OF TUMOURS OF THE MOUSE BLADDER EPITHELIUM BY 4-ETHYLSULPHONYLNAPHTHALENE-1-SULPHONAMIDE

D. B. CLAYSON AND G. M. BONSER

*From the Department of Experimental Pathology and Cancer Research, The School of Medicine, Leeds, 2*

Received for publication January 22, 1965

The demonstration that 4-ethylsulphonylnaphthalene-1-sulphonamide (HPA) induces hyperplasia of the bladder epithelium in the rat and the mouse (Paget, 1958 ; Sen Gupta, 1962*a*) suggested that the chemical might act either as a cocarcinogen or as a promoting agent to this tissue. Such properties would be valuable in designing experiments to elucidate the mechanism of induction of bladder tumours and, accordingly, two series of experiments were started.

Bonser and Clayson (1964) showed that HPA was carcinogenic to the bladder epithelium of hybrid Ab × IF mice when it was incorporated at a level of 0·01 per cent in the diet for up to 65 weeks. Females were more susceptible than males. The incidence of tumours was not increased by the administration of a single oral dose of 2·5 mg. 9,10-dimethyl-1,2-benzanthracene (DMBA) one week before the start of treatment with HPA.

Sen Gupta (1962*b*) implanted paraffin wax pellets containing 2-amino-1-naphthol hydrochloride into the lumen of the bladder of stock mice, and found that the oral administration of HPA considerably augmented the incidence of tumours. Unfortunately he did not determine the influence of HPA on the tumour yield in mice implanted with unadulterated paraffin wax pellets. Therefore it is not possible to decide, on the basis of this experiment alone, whether the HPA acted as a cocarcinogen or whether it was a more potent complete carcinogen when it acted on the bladders of mice containing a foreign body, i.e. the pellet.

In this report further evidence is given on the possible promoting or cocarcinogenic actions of HPA.

### MATERIALS AND METHODS

F1 hybrid mice of strains C57 and IF, and Ab and IF were bred in the laboratory. They received Oxo Diet 41B and water *ad libitum*, and were used for experiment when they were approximately 10 weeks of age.

Bladder implantation was carried out in female C57 × IF mice by the method of Jull (1951) as modified by Allen, Boyland, Dukes, Horning and Watson (1957). Bladders were prepared for histology as described by Sen Gupta (1962*a*).

4-Ethylsulphonylnaphthalene-1-sulphonamide (HPA) was prepared from naphthionic acid by the method of Brimelow and Vasey (1958). Chromatographically purified 1-phenylazo-2-naphthol and crushed paraffin wax were obtained from the British Drug Houses Ltd., and 2-acetamidofluorene (AAF) and 9,10-dimethyl-1,2-benzanthracene (DMBA) from L. Light & Co. Ltd.

TABLE I.—*Ab × IF Mice Treated with HPA (0·01 per cent of Diet) With and Without Other Chemicals*

| | | | Number of mice dying within stated period (weeks) | | | | | | Bladder lesions | | | | | | |
|---|---|---|---|---|---|---|---|---|---|---|---|---|---|---|---|
| | | | | | | | | | | Squamous | | Carcinoma† | | | |
| Experiment | Treatment‡ | Sex | 30–39 | 40–49 | 50–59 | 60–69* | Total | Hepatomas | Hyperplasia | metaplasia | Papilloma† | I | II | III | Total |
| 1§ | HPA only | M. | 2 | 4 | 1 | 9 | 16 | 0 | 11 | 4 | 1 | 1 | 1 | 0 | 2 |
| 2§ | HPA only | F. | 2 | 4 | 3 | 10 | 19 | 0 | 11 | 8 | 1 | 6 | 3 | 0 | 9 |
| 3§ | DMBA + HPA | M. | 0 | 6 | 4 | 7 | 17 | 0 | 10 | 3 | 0 | 3 | 1 | 0 | 4 |
| 4§ | DMBA + HPA | F. | 1 | 4 | 6 | 7 | 18 | 0 | 14 | 6 | 4 | 4 | 0 | 1 | 5 |
| 5 | HPA only | M. | 0 | 0 | 6 | 12 | 18 | 0 | 17 | 0 | 1 | 2 | 1 | 0 | 3 |
| 6 | HPA only | F. | 0 | 0 | 1 | 19 | 20 | 0 | 14 | 4 | 2 | 5 | 3 | 1 | 9 |
| 7 | AAF + HPA | M. | 0 | 0 | 0 | 9 | 9 | 0 | 5 | 1 | 0 | 0 | 0 | 0 | 0 |
| 8 | AAF + HPA | F. | 0 | 1 | 1 | 16 | 18 | 0 | 14 | 5 | 5 | 3 | 3 | 0 | 6 |
| 9 | AAF only | M. | 0 | 0 | 3 | 16 | 19 | 6 | 2 | 0 | 0 | 0 | 0 | 0 | 0 |
| 10 | AAF only | F. | 0 | 0 | 0 | 14 | 14 | 1 | 1 | 0 | 0 | 0 | 0 | 0 | 0 |

* all surviving mice were killed at 65 weeks except 4 in experiment 1, which were killed at 69 weeks.
† only the most advanced lesion is recorded.
‡ for details see text.
§ Bonser and Clayson (1964).

Probabilities were evaluated by the exact method for 2 × 2 tables (Fisher, 1950).

### RESULTS

### *The oral administration of HPA to Ab × IF mice*

Experiments 1–4 (Table I) are those described by Bonser and Clayson (1964). A small number of mice have been added to those originally recorded and two which failed to live for 30 weeks have been excluded. When HPA was administered at a level of 0·01 per cent in the diet (experiments 1, 2), 9 out of 19 female and 2 out of 16 male Ab × IF mice developed carcinomas, 7 Grade I (that is to say, histologically malignant but not invading muscle) and 4 Grade II (into muscle). There is a significant difference in carcinoma incidence between the sexes (P = 0·015). The administration of a single dose of 2·5 mg. DMBA one week before the start of treatment with HPA (experiments, 3, 4) diminished the yield of carcinomas in female mice but had little effect on the combined incidence of papillomas and carcinomas. Whereas the combined tumour incidence was in favour of the females, the carcinomas were fairly evenly divided between the sexes.

It was decided to try to confirm the sex differences in the incidence of carcinomas induced by HPA alone and to determine whether another form of pretreatment would augment the yield of tumours. AAF was chosen for the pretreatment because, unlike systemically administered DMBA, it is *known* to be carcinogenic to the mouse bladder (Armstrong and Bonser, 1947; Foulds, 1947). Three groups of male and three of female Ab × IF mice (experiments 5–10) were set up. One group of male and one of female mice were given 0·01 per cent of HPA for the duration of the experiment. The remainder were given 0·03 per cent AAF in the diet for 4 weeks only and one group of males and one of females were subsequently left untreated while the others were transferred to the HPA diet.

The carcinogenic activity of HPA to the bladder epithelium was confirmed. There were 3 carcinomas in 18 male and 9 carcinomas in 20 female mice. Seven of these were Grade I, 4 were Grade II and one was Grade III (that is to say, had spread through the bladder wall). The female was again more susceptible than the male although the difference did not attain statistical significance ($P = 0{\cdot}061$). The overall incidence of bladder tumours in female Ab × IF mice treated with HPA in the diet was 46 per cent (18 carcinomas in 39 mice) and in males was 15 per cent (5 carcinomas in 34 mice). This difference is statistically significant ($P = 0{\cdot}0037$).

Pretreatment with AAF reduced rather than augmented the yield of carcinomas. No carcinomas were found in 9 male, and 6 carcinomas in 20 female mice which had received both AAF and HPA ($P = 0{\cdot}063$). In addition 5 female mice had papillomas but no carcinomas. Therefore it appears that the administration of DMBA or AAF reduces the induction of frank carcinomas in female mice by HPA. The effect in male mice is not clear because of the small number of tumours.

In only 3 of 33 mice treated with AAF for one month, and then left for the duration of the experiment, was hyperplasia of the bladder present. No other bladder lesions were found. In 6 of 19 male and one of 14 female mice treated in this way there were hepatomas. Similar tumours occur spontaneously in Ab × IF mice more than 80 weeks of age (Clayson, Lawson, Santana and Bonser, 1965), the incidence being 40 per cent in males and 14 per cent in females. The limited dose of AAF may have reduced the latent period of these tumours to a certain extent. Because the number of male mice surviving treatment with AAF and HPA was small (experiment 7) it is not possible to be categorical about the inhibition of the liver tumours by HPA. However, fatty degeneration and centrilobular necrosis were found in about one quarter of all the mice treated with AAF alone and were not observed in those mice treated with AAF and HPA. Therefore we consider that the liver lesions may have been inhibited by HPA in the doubly treated mice.

*The effect of oral HPA on C57 × IF mice with pellets in the bladder*

Sen Gupta (1962*b*) showed that orally administered HPA increased the incidence of tumours induced by the implantation of pellets containing 2-amino-1-naphthol hydrochloride into the lumen of the bladder of stock mice. In the present series of experiments it was decided to use a more stable carcinogen, 1-phenylazo-2-naphthol, in place of the labile 2-amino-1-naphthol hydrochloride. Approximately 200 female C57 × IF mice were implanted with 15–17 mg. pellets of crushed paraffin wax and a further 200 with similar pellets containing 12·5 per cent of the azo compound. Half of the mice in each group were given no further treatment (experiments 11, 13) and, after 2 weeks had elapsed to allow the effects of the operation to subside, the remainder were given a diet containing 0·005 per cent HPA (experiments 12, 14). All surviving mice were killed 40 weeks after the operation.

Carcinomas were found in all four groups of mice (Table II). The incidence in mice implanted with paraffin wax only was 4·5 per cent (4 carcinomas in 89 mice) which was raised to 19 per cent (14 carcinomas in 75 mice) by the oral administration of HPA. Pellets containing 1-phenylazo-2-naphthol induced a 14 per cent incidence of tumours (14 carcinomas in 100 mice) and this was increased substantially by HPA to 73 per cent (44 carcinomas in 60 mice). The incidence of tumours induced by 1-phenylazo-2-naphthol alone was considerably lower than

TABLE II.—*The Influence of Orally Administered HPA on the Incidence of Epithelial Tumours in C57 × IF Mice Implanted with Pellets in the Bladder*

| Experiment | Nature of pellets | Oral administration of HPA (0·005 per cent) | Number of mice surviving 25 weeks | Squamous metaplasia | Papillomas or adenomas | Carcinomas | | | | |
|---|---|---|---|---|---|---|---|---|---|---|
| | | | | | | I | II | III | Total | Per cent |
| 11 | Paraffin wax | — | 89 | 0 | 0 | 3 | 1 | 0 | 4 | 4·5 |
| 12 | Paraffin wax | + | 75 | 14 | 2 | 11 | 3 | 0 | 14 | 18·7 |
| 13 | 1-Phenylazo-2-naphthol | — | 100 | 2 | 0 | 9 | 5 | 0 | 14 | 14·0 |
| 14 | 1-Phenylazo-2-naphthol | + | 60 | 16 | 0 | 25 | 19 | 0 | 44 | 73·3 |

TABLE III.—*The Carcinogenic Activity of HPA After Bladder Implantation in C57 × IF Mice*

| Experiment | Nature of pellets | Number of mice surviving 25 weeks | Squamous metaplasia | Papillomas or adenomas | Carcinomas | | | | | |
|---|---|---|---|---|---|---|---|---|---|---|
| | | | | | I | II | III | Total | Per cent | Probability |
| 11 | Paraffin wax only | 89 | 0 | 0 | 3 | 1 | 0 | 4 | 4·5 | — |
| 15 | HPA in wax | 37 | 0 | 1 | 6 | 2 | 0 | 8 | 21·6 | 0·0056 |

the 25 per cent obtained in a previous experiment (Bonser, Bradshaw, Clayson and Jull, 1956).

The bladder epithelium of mice treated with HPA showed a greater tendency to squamous metaplasia than that of those which only had pellets in the bladder. The carcinomas, however, were all of Grade I or Grade II and no trend to greater malignancy with increasing tumour yield was apparent.

*The bladder implantation of HPA*

Although the induction of bladder tumours following the systemic administration of an otherwise innocuous chemical has previously been taken to indicate that the chemical is active by virtue of a metabolic conversion, it was decided to investigate HPA for local carcinogenic activity by bladder implantation. It was found (Table III) that HPA in a 12·5 per cent suspension in crushed paraffin wax induced 22 per cent of carcinomas (8 tumours in 37 mice) compared to a 4·5 per cent incidence in the controls. It is thus locally active to the bladder epithelium of the mouse (P = 0·0056).

### DISCUSSION

4-Ethylsulphonylnaphthalene-1-sulphonamide (HPA) does not appear to be related to any of the known groups of chemical carcinogens. One other sulphonamide has been reported in the literature to induce carcinomas of the bladder in the rat (Hueper, 1962). The investigations of HPA have, so far, shown that it induces cancer of the bladder epithelium in hybrid Ab × IF mice following systemic administration. Its long term effects in other mice and in other species remain to be examined.

The greater susceptibility of the female Ab × IF mouse to the induction of bladder cancer by HPA, compared with the male, may have one of the two following explanations. It is possible that mouse bladder epithelium is under direct hormonal control and, when the correct carcinogenic stimulus is applied, in the female it progresses more easily to malignancy. However, Armstrong and Bonser (1947) found that in the RIII, IF and White Label strains the male mouse was slightly more susceptible to the induction of bladder tumours by AAF than was the female. Foulds (1947) who treated RIII mice with the same carcinogen for a shorter period, showed that the male mice developed bladder cancer in 12 out of 23 cases whereas none of the 13 females did so. Therefore, if hormones are thought to influence directly the genesis of bladder tumours, the male environment must favour the induction of tumours by AAF and the female environment those induced by HPA.

The second possible reason for the sex differences in the incidence of bladder cancer is that the hormonal environment affects the metabolism of these chemicals and that the female excretes more of the locally active metabolite of HPA into the urine whereas the male excretes more of that of AAF. Weisburger, Grantham and Weisburger (1964) examined the metabolism of the suspected locally active metabolite of AAF, *N*-hydroxy-2-acetamidofluorene, in Fischer rats and found that the female excreted considerably more of this compound in the urine than did the male. Intraperitoneal injection of AAF itself into male and female ACI rats showed similar but less marked differences in the pattern of metabolites (Weisburger and Weisburger, 1963). Further studies along these lines are needed.

The considerable incidence of bladder carcinomas obtained after the oral administration of HPA indicates that it is a complete carcinogen and not solely a promoting agent, which should ideally induce cancers only after the application of an initiating agent. The fact that the prior oral administration of two potent carcinogens, AAF and DMBA, did not increase the tumour yield supports the view that HPA is a complete carcinogen. The diminution in incidence of carcinomas in females following the application of AAF or DMBA is possibly due to the effect of these chemicals on the metabolism of HPA.

Sen Gupta's (1962*b*) observation that oral HPA increases the incidence of tumours induced by pellets implanted into the bladder has been confirmed. Oral HPA increases the tumour yield with paraffin wax pellets from 4·5 to 19 per cent and with pellets containing 1-phenylazo-2-naphthol from 14 to 73 per cent. It is interesting, although it may be coincidental, that the ratios of the tumour incidences with and without HPA are similar for the paraffin wax pellets (4·2) and for those containing 1-phenylazo-2-naphthol (5·2).

Relatively little is known about the mechanism of induction of either hyperplasia or carcinoma of the bladder by HPA. By analogy with the aromatic amines (Clayson, 1964) it was suspected that HPA would be effective by virtue of its conversion to an active metabolite excreted by way of the urine (Santana, 1963). However, it has been found that HPA itself is locally active to the bladder epithelium when tested by bladder implantation. Therefore if, as seems plausible from the attempts to account for the results of the other experiments, the carcinogenic activity of HPA depends on its metabolism, it follows that *either* HPA must be converted *in vivo* to a more active substance *or* that it is metabolised to an easily excretable form which is re-converted in the urinary tract or its epithelium to HPA itself.

### SUMMARY

1. 4-Ethylsulphonylnaphthalene-1-sulphonamide is carcinogenic to the bladder epithelium of the Ab $\times$ IF mouse after oral administration. The incidence of tumours in female mice (46 per cent) was greater than in male mice (15 per cent).

2. The administration of 9,10-dimethyl-1,2-benzanthracene or 2-acetamidofluorene before 4-ethylsulphonylnaphthalene-1-sulphonamide depressed the incidence of carcinomas in female mice but had little effect in the male.

3. Oral 4-ethylsulphonylnaphthalene-1-sulphonamide increased the incidence of carcinomas induced by the bladder implantation of plain paraffin wax pellets or those containing 1-phenylazo-2-naphthol four- to five-fold.

4. 4-Ethylsulphonylnaphthalene-1-sulphonamide was incorporated into paraffin wax pellets and implanted in the lumen of the mouse bladder. The incidence of carcinomas was significantly increased over that induced by paraffin wax alone. It is judged to be a locally active carcinogen to the bladder epithelium.

5. These results are discussed in the light of the possible promoting and cocarcinogenic properties of the chemical. It is suggested that variation in the metabolism of the compound may offer the best explanation of the results.

We thank Mrs. Doris Khan and Miss Margery Wood, B.Sc., for operating upon and caring for the bladder implanted mice.

### REFERENCES

Allen, M. J., Boyland, E., Dukes, C. E., Horning, E. S. and Watson, J. G.—(1957) *Brit. J. Cancer*, **11**, 212.

Armstrong, E. C. and Bonser, G. M.—(1947) *J. Path. Bact.*, **59**, 19.

Bonser, G. M., Bradshaw, L., Clayson, D. B. and Jull, J. W.—(1956) *Brit. J. Cancer*, **10**, 539.

*Idem* and Clayson, D. B.—(1964) *Brit. J. Urol.*, **36**, 26.

Brimelow, H. C. and Vasey, C. H.—(1958) *U.K. Patent* No. 791, 529.

Clayson, D. B.—(1964) *Brit. med. Bull.*, **20**, 115.

*Idem*, Lawson, T. A., Santana, S. and Bonser, G. M.—(1965) *Brit. J. Cancer*, **19**, 297.

Fisher, R. A.—(1950) 'Statistical Methods for Research Workers'. 11th Edition. Edinburgh (Oliver and Boyd).

Foulds, L.—(1947) *Brit. J. Cancer*, **1**, 172.

Hueper, W. C.—(1962) *Clin. Pharmacol. Ther.*, **3**, 776.

Jull, J. W.—(1951) *Brit. J. Cancer*, **5**, 328.

Paget, G. E.—(1958) 'A Symposium on the Evaluation of Drug Toxicity'. Edited by A. L. Walpole and A. Spinks. London (J. & A. Churchill).

Santana, S.—(1963) *Brit. J. Cancer*, **17**, 715.

Sen Gupta, K. P.—(1962*a*) *Ibid.*, **16**, 110.—(1962*b*) *Nature, Lond.*, **194**, 1185.

Weisburger, E. K., Grantham, P. H. and Weisburger, J. H.—(1964) *Biochemistry*, **3**, 808.

Weisburger, J. H. and Weisburger, E. K.—(1963) *Acta Un. int. Cancr.*, **19**, 513.